# OSMR deficiency aggravates pressure overload-induced cardiac hypertrophy by modulating macrophages and OSM/LIFR/STAT3 signalling

**DOI:** 10.1186/s12967-023-04163-x

**Published:** 2023-04-29

**Authors:** Yizhou Feng, Yuan Yuan, Hongxia Xia, Zhaopeng Wang, Yan Che, Zhefu Hu, Jiangyang Deng, Fangfang Li, Qingqing Wu, Zhouyan Bian, Heng Zhou, Difei Shen, Qizhu Tang

**Affiliations:** 1 Department of Cardiology, Renmin Hospital of Wuhan University, Wuhan, 430060 China; 2 Hubei Key Laboratory of Metabolic and Chronic Diseases, Wuhan, 430060 China; 3 Cardiovascular Research Institute of Wuhan University, Wuhan, 430060 China

**Keywords:** Heart failure, Hypertrophy, OSMR, LIFR, Macrophage

## Abstract

**Background:**

Oncostatin M (OSM) is a secreted cytokine of the interleukin (IL)-6 family that induces biological effects by activating functional receptor complexes of the common signal transducing component glycoprotein 130 (gp130) and OSM receptor β (OSMR) or leukaemia inhibitory factor receptor (LIFR), which are mainly involved in chronic inflammatory and cardiovascular diseases. The effect and underlying mechanism of OSM/OSMR/LIFR on the development of cardiac hypertrophy remains unclear.

**Methods and results:**

OSMR-knockout (OSMR-KO) mice were subjected to aortic banding (AB) surgery to establish a model of pressure overload-induced cardiac hypertrophy. Echocardiographic, histological, biochemical and immunological analyses of the myocardium and the adoptive transfer of bone marrow-derived macrophages (BMDMs) were conducted for in vivo studies. BMDMs were isolated and stimulated with lipopolysaccharide (LPS) for the in vitro study. OSMR deficiency aggravated cardiac hypertrophy, fibrotic remodelling and cardiac dysfunction after AB surgery in mice. Mechanistically, the loss of OSMR activated OSM/LIFR/STAT3 signalling and promoted a proresolving macrophage phenotype that exacerbated inflammation and impaired cardiac repair during remodelling. In addition, adoptive transfer of OSMR-KO BMDMs to WT mice after AB surgery resulted in a consistent hypertrophic phenotype. Moreover, knockdown of LIFR in myocardial tissue with Ad-shLIFR ameliorated the effects of OSMR deletion on the phenotype and STAT3 activation.

**Conclusions:**

OSMR deficiency aggravated pressure overload-induced cardiac hypertrophy by modulating macrophages and OSM/LIFR/STAT3 signalling, which provided evidence that OSMR might be an attractive target for treating pathological cardiac hypertrophy and heart failure.

**Supplementary Information:**

The online version contains supplementary material available at 10.1186/s12967-023-04163-x.

## Introduction

Cardiac remodelling is an important pathophysiological process in the progression of various cardiovascular diseases into heart failure, but the specific mechanism has not been fully determined [1]. Pressure overload-induced cardiac remodelling manifests at the cellular and molecular levels as myocyte hypertrophy, cell death, interstitial fibrosis, inflammatory responses and metabolic disorders [2, 3]. These events adaptively protect the heart against pathological stresses in the early stage; however, persistent uncontrolled pathological pressure overload leads to these remodelling events becoming maladaptive and predisposing the individual to cardiovascular morbidity and mortality. Preventing and reversing adverse myocardial remodelling is critical for improving the prognosis of heart failure (HF) and has become an important therapeutic target for HF [4].

Macrophages constitute the main part of mammalian heart tissue and play a crucial role in the development and progression of cardiac remodelling. Accordingly, under a variety of internal and external stimuli, macrophages may generate and secrete cytokines through the activation of intracellular signalling pathways, affecting their proliferation, migration, transformation and secretion of cytokines, which in turn mediate cardiomyocyte hypertrophy, cardiac fibroblast proliferation, transdifferentiation and collagen production during the occurrence and development of cardiac remodelling [5, 6]. Macrophage depletion has been reported to attenuate the adverse cardiac remodelling and fibrosis induced by pressure overload [7]. In mouse heart tissue after Ang II perfusion, diverse subtypes of cardiac macrophages are expanded, including the proliferation of resident macrophages in heart tissue and the recruitment of Ly6C^+^ monocytes from the blood [5]. Therefore, targeting the regulation of macrophages might provide promising strategies for delaying and reversing adverse cardiac remodelling.

Oncostatin M (OSM) is a member of the interleukin (IL)-6 family and interacts with the functional receptor complexes of shared the signal transducing component glycoprotein 130 (gp130) and OSM receptor β (OSMR) or leukaemia inhibitory factor receptor (LIFR), which functions as a key regulator of communication in target cells in inflammatory and various cardiovascular diseases, including aortic stenosis, myocardial infarction, myocarditis, cardiac sarcoidosis, and various cardiomyopathies [8]. OSM secretion is increased when neutrophils, CD4^+^ T cells and monocytes/macrophages are stimulated, which causes changes in endothelial cell and smooth muscle cell proliferation, age-related obesity and a reduction in insulin resistance in tissues and organs [9–11]. Recently, OSM has gained attention as a major mediator of cardiomyocyte dedifferentiation and remodelling under pathological conditions [12–14]. In a mouse model of myocardial infarction, short-term activation of OSM signalling can promote wound healing, while sustained high levels of OSM can promote the development of dilated cardiomyopathy and heart failure [13]. Knockout of the OSMR gene in mice exacerbates high-fat diet-induced obesity and leads to adipose tissue inflammation and insulin resistance by modulating the function of macrophages [15, 16]. In the present study, we found that the expression of OSMR was significantly increased in murine hypertrophic hearts induced by pressure overload. Moreover, the expression of OSMR mRNA was significantly decreased in macrophages but did not change significantly in cardiomyocytes or cardiac fibroblasts after stimulation with Ang II (Additional file 1: Fig. S1). This evidence suggests that OSM/OSMR signalling is involved in cardiac remodelling, which we hypothesized was mediated by inflammation and macrophages. In this study, we used C57BL/6J mice and OSMR global knockout mice to test our hypothesis and explore the functional role of OSMR in pressure overload-induced cardiac remodelling and the underlying mechanism.

## Methods

### Animals and animal models

All of the animal experiments complied with the Guidelines for the Care and Use of Laboratory Animals published by the United States National Institutes of Health (NIH Publication, revised 2011) and were approved by the Laboratory Animal Welfare & Ethics Committee of Renmin Hospital of Wuhan University. Male OSMR-knockout (OSMR-KO) and wild-type (WT) C57BL/6J mice (8–10 weeks old, 23.5–27.5 g) were used in our study. OSMR-KO mice were on the C57BL/6J background and donated by RIKEN BioResource Center (RBRC 02711) [17]. C57BL/6J mice were purchased from the Institute of Laboratory Animal Science at the Chinese Academy of Medical Sciences (Beijing, China).

Male Osmr-KO and WT control mice were subjected to aortic banding (AB) surgery or sham operation as described previously [18]. The mice were anaesthetized with 3% pentobarbital sodium solution (#P3761, Sigma‒Aldrich, USA) (80 mg/kg, ip) and then intubated and mechanically ventilated under pressure mode with a pressure of 13–15 cmH_2_O and a breathing rate of 105 bpm. After the skin was disinfected, an incision (approximately 5 mm) was made horizontally along the left 2–3 intercostal space, and the muscle and soft tissue were separated successively. The intercostal muscle was then separated at the left margin 2 mm from the sternum at the 2–3 intercostal space, and the chest cavity was opened with a chest expander by approximately 5 mm. The descending branch of the aortic arch was isolated. Surgical sutures (7-0) were threaded through the thoracic aorta, and the pad needle was placed parallel to the aorta. After a knot was made, the pad needle was quickly removed to complete aortic coarctation, and the chest and skin were closed; the mice were allowed to recover. In the sham operation group, the mice underwent the same procedure without ligation.

All mice were subjected to echocardiographic and haemodynamic measurements at 2, 4, and 8 weeks postoperation and were then euthanized, and materials for histopathological, molecular analyses and flow cytometry analyses were collected according to the local relevant guidelines. All operations and experimental data analyses were performed and processed in a blinded manner.

### Echocardiography and haemodynamics

Transthoracic echocardiography was performed on mice at the indicated times after sham or AB surgery to evaluate cardiac structure and function by using a Mylab 30CV (Esaote S.P.A., Genoa, Italy) equipped with a 10-MHz linear-array ultrasound transducer as previously described [19]. The mice were initially anaesthetized with 1.5% isoflurane and then placed in the left decubitus position. The left ventricle was assessed in the parasternal short-axis view at a frame rate of 120 Hz. The parameters, including left ventricular end-diastolic diameter (LVEDd), left ventricular end-systolic diameter (LVESd), left ventricular end-diastolic posterior wall thickness (LVPWd), interventricular septal thickness at diastole (IVSd), left ventricular ejection fraction (LVEF) and fractional shortening (FS), were measured and assessed by M-mode tracings with a sweep speed of 50 mm/s at the mid-papillary muscle level.

In vivo haemodynamic analysis was performed in the chest-closed, 1.5% isoflurane-anaesthetized mouse by cardiac catheterization. A microtip catheter transducer (SPR-839, Millar Instruments, Houston, Texas, USA) was placed through the right carotid artery and positioned along the longitudinal axis of the left ventricle. After stabilization for 15 min, the pressure signals and heart rate were recorded continuously with an ARIA pressure‒volume conductance system coupled with a Powerlab/4SP A/D converter and then stored and displayed on a personal computer as described previously [18]. The data were processed and analysed using LabChart 7 software (AD Instruments, CO, USA).

### Histological analysis

Hearts were excised, immediately placed in 10% potassium chloride solution to ensure that they were stopped in diastole and fixed with 10% neutral paraformaldehyde for at least 24 h. The hearts were dissected transversely from the mid-papillary level, dehydrated with graded concentrations of dimethylbenzene-alcohol, embedded in paraffin, and subsequently cut into 4–5 μm-thick slices. The sections were subjected to haematoxylin and eosin (HE) staining for morphometric analyses or picrosirius red staining (PSR) to evaluate fibrosis in the heart. Myocyte cross-sectional areas (CSAs) were determined in more than 100 cells per group, and the fibrotic areas were quantified in at least 60 fields of view (200× magnification) per group. For immunofluorescence analysis, the heart tissue sections were incubated with primary antibodies against OSM (#sc-374039, Santa Cruz, USA)/LIFR (#22779-1-AP, Proteintech, China) and α-Actinin (#ab90421, Abcam, USA) at 4 °C overnight. The next day, the sections were incubated with Alexa Fluor® 488 donkey anti-mouse/rabbit (#R37114/#A21206, Invitrogen, USA) and Alexa Fluor® 568 donkey anti-mouse secondary antibodies (#A10037, Invitrogen, USA). Nuclei were stained with DAPI (#S36939, Invitrogen, USA). Images were obtained by a Nikon H550L microscope (Tokyo, Japan) or Olympus DX51 fluorescence microscope (Tokyo, Japan) and analysed using digital image analysis software (Image-Pro Plus 6.0, Media Cybernetics, Bethesda, USA).

### Protein extraction and western blot analysis

Cardiac tissues or cells were lysed in RIPA lysis buffer, and protein concentrations were determined with a BCA protein assay kit (#23227, ThermoFisher Scientific, USA) and ELISA reader (Synergy HT, BioTek, USA). Protein lysates were loaded into 10% SDS‒PAGE gels for electrophoresis and then transferred onto polyvinyl difluoride (PVDF) membranes (#IPFL00010, Millipore, USA). After being blocked in 5% skim milk at room temperature for 1 h, the PVDF membranes were incubated with primary antibodies (Additional file 1: Table S1) at 4 °C overnight and with secondary antibodies for 1 h at room temperature. The blots were detected using a chemiluminescence ECL kit (#1705062, Bio-Rad, USA) and scanned by ImageLab software (v5.2.1, Bio-Rad, USA). Target protein expression levels were normalized to glyceraldehyde-3-phosphate dehydrogenase (GAPDH).

### RNA isolation and quantitative real-time PCR (RT‒PCR)

Total RNA was isolated from frozen myocardium or cultured cells using TRIzol reagent (#15596-026, ThermoFisher Scientific, USA). Two micrograms of RNA was reverse transcribed into cDNA using the Transcriptor First Strand cDNA Synthesis Kit (#4896866001, Roche, Switzerland) according to the manufacturer’s protocol. Then, quantitative real-time PCR was performed using LightCycler 480 SYBR Green 1 Master Mix (#04707516001, Roche, Switzerland) and a LightCycler 480 qPCR System (Roche, Switzerland); the primers are shown in Additional file 1: Table S2. mRNA expression levels were normalized to GAPDH.

### Bone marrow-derived macrophage (BMDM) isolation and culture

Bone marrow cells from WT and OSMR-KO mice were isolated and cultured as described previously [20]. Briefly, the femur and tibia were removed from the mice under sterile conditions. Bone marrow cells were isolated from murine femurs and tibias, cultured in RPMI 1640 medium (#11875-093, Gibco, USA) supplemented with 10% foetal bovine serum (FBS) (#086-150, Wisent, USA) and the antibiotics penicillin/streptomycin (#15140-122, Gibco, USA) after being lysed with red blood cell lysis buffer (#11814389001, Sigma‒Aldrich, USA) and incubated at 5% CO_2_ and 37 °C. To differentiate BMDMs, 50 ng/mL M-CSF (#SRP3221, Sigma‒Aldrich, USA) was added to the medium on the first and 3 days of in vitro culture. Bone marrow macrophages were fully differentiated after 5–7 days in culture and then harvested for follow-up experiments.

### Immunofluorescence staining

To stain F4/80 and LIFR, BMDMs from OSMR-KO and WT mice were cultured on coverslips, stimulated with 100 ng/mL LPS (#L4391, Sigma‒Aldrich, USA) or PBS for 24 h after serum starvation for 12 h, fixed with 4% paraformaldehyde, permeabilized in 0.5% Triton X-100, and incubated with anti-F4/80 (#ab6640, Abcam, USA) and anti-LIFR (#22779-1-AP, Proteintech, China) antibodies at 4 °C overnight. The next day, the cells were washed and incubated with Alexa Fluor^®^ 568 goat anti-rat or Alexa Fluor^®^ 488 goat anti-rabbit secondary antibodies (#A-11077, #A32731, Invitrogen, USA) for 60 min at 37 °C. Nuclei were stained with DAPI (#S36939, Invitrogen, USA). The slides were observed and photographed using an Olympus DX51 fluorescence microscope (Tokyo, Japan) and analysed using Image-Pro Plus 6.0 software (Media Cybernetics, Bethesda, USA).

### Sample preparation and flow cytometry

To examine monocytes/macrophages and their subsets, peripheral blood was collected in BD vacutainer K2EDTA (EDTA) tubes (#367841, BD Bioscience, USA) on the day of sacrifice. The samples were then incubated with fluorescence-conjugated antibodies at room temperature for 20 min in the dark, lysed with red blood cell lysis buffer (#11814389001, Sigma‒Aldrich, USA) and washed with cell staining buffer (#420201, BioLegend, USA) for flow cytometric analysis.

To examine immune cells in the heart, whole ventricles were quickly excised, minced and enzymatically digested with type II collagenase (#LS004176, Worthington, USA) and collagenase/dispase (#10269638001, Roche, Switzerland) in a 37 °C water bath with gentle agitation. Single cells from cardiac tissues were passed through a 100 μm strainer with 10 ml of cold buffer (PBS + 0.5% BSA + 2 mM EDTA). After erythrocyte lysis using red blood cell lysis buffer (#11814389001, Sigma‒Aldrich, USA), the cells were counted using an automated cell counter (Countess, Invitrogen, USA). Ten million cells from single-cell suspensions were incubated with anti-CD16/32 (#101302, BioLegend, USA) antibodies at 4 °C for 10 min to block nonspecific binding, followed by staining with fluorescence-conjugated antibodies in the dark (Additional file 1: Table S1). Dead cells were stained with 7-AAD Viability Staining Solution (#420404, BioLegend, USA) 5 min before the assay.

Data were acquired on a BD FACS Arial II flow cytometer (BD Biosciences, USA) equipped with 488 nm, 640 nm, 375 nm and 561 nm excitation lasers. The data were analysed using FlowJo software (TreeStar, USA) and are expressed as the number of positive cells per heart.

### Adoptive transfer of BMDMs

Bone marrow cells were isolated and cultured as described above. Adherent macrophages were harvested after 7 days in culture. BMDMs (2 × 10^7^) were obtained from WT or OSMR-KO mice or PBS and adoptively transferred via the tail vein into WT mice 3 days after AB surgery. Two weeks later, echocardiography and haemodynamics were performed, and the hearts, lungs and tibiae of the sacrificed mice were dissected for further analyses.

### Intramyocardial injection

To specifically block LIFR expression in cardiac tissue, adenovirus-mediated intramyocardial short hairpin RNA (shRNA) transfer was performed [21]. In brief, a horizontal skin incision at the level of the 2–3 intercostal space was made on anaesthetized and mechanically ventilated mice, and AB surgery was performed. After stitching the incision at the 2–3 intercostal space, the 3–4 intercostal space was opened, and the heart was smoothly and gently “popped out”, followed by direct intramyocardial injection of Ad-shRNA-LIFR or Ad-shRNA (1 × 10^9^ pfu) in the left ventricular free wall (1 × 10^11^ pfu, 10 µl) with a microsyringe equipped with a 31-gauge needle. After injection, the heart was immediately placed back, followed by manual evacuation of pneumothoraces, sternal closure and skin suturing, and the mice were allowed to recover. All mice were euthanized 4 weeks after surgery to evaluate the phenotype.

The recombinant adenovirus Ad-shRNA-LIFR carrying a shRNA sequence targeting LIFR (5’-GCCACATCATTCTGACATCAA-3’) and empty control virus Ad-shRNA were constructed and produced by Hanbio Company (Shanghai, China).

### Statistical analyses

The data are presented as the mean ± SEM. All calculations and statistics were performed with GraphPad Prism 8.4.2 (GraphPad Prism Software Inc., San Diego, CA, USA). Data distribution was tested for normality using a Kolmogorov‒Smirnov normality test. Nonnormally distributed data were analysed using nonparametric tests (Mann‒Whitney test for 2 groups and Kruskal‒Wallis test for > 2 groups). For data that followed a normal distribution, unpaired Student’s *t* test was used for comparisons between two groups, while differences between multiple groups were analysed using one-way or two-way analysis of variance (ANOVA) followed by the Bonferroni posttest. Kaplan‒Meier curves and log-rank tests were used to compare survival. Values of P < 0.05 were considered statistically significant.

## Results

### OSMR deficiency aggravates cardiac hypertrophy and reduces survival in mice with pressure overload-induced hypertrophy

**Fig. 1:**
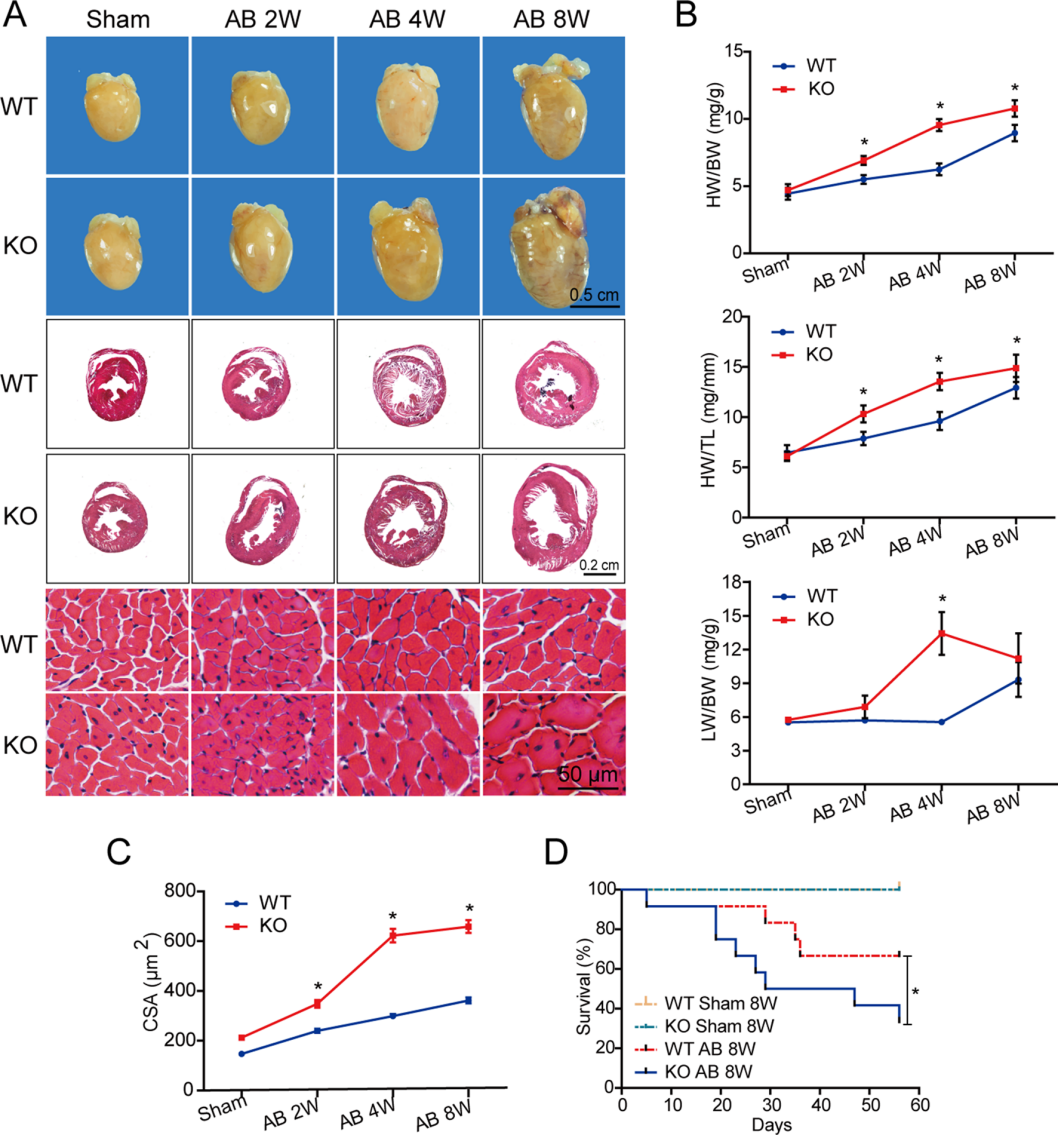
OSMR deficiency aggravates pressure overload-induced cardiac hypertrophy and reduces survival. **A** Representative gross morphology and haematoxylin and eosin-stained heart sections of wild-type (WT) or OSMR-KO mice subjected to sham or AB surgery at the indicated times (n = 8 mice per experimental group). **B** Heart weight to body weight (HW/BW) ratios, lung weight to body weight (LW/BW) ratios and heart weight to tibia length (HW/TL) ratios of WT or OSMR-KO mice at the indicated times (n = 8 mice per experimental group). **C** Quantification of the cross-sectional area in each group (n = 100–200 cells per experimental group). **D** Survival rates of mice in each group at eight weeks after sham or AB surgery (n = 12 mice per experimental group). Data are presented as the mean ± SEM. *P < 0.05 compared with the matched control

To investigate the specific effect of OSMR on pressure overload-induced cardiac hypertrophy, we used OSMR whole-body knockout mice (Additional file 1: Fig. S2). These mice and age-matched wild-type controls were subjected to AB surgery for 2, 4 and 8 weeks. Although no notable differences were observed at the basal level, the aggravation of hypertrophy in OSMR-KO hearts was confirmed by ventricular enlargement and cardiac hypertrophy in gross heart morphology, larger individual cardiomyocyte cross-sectional areas and increased ratios of heart weight to body weight or tibia length at 2, 4, and 8 weeks after AB surgery (Fig. 1A–C). The ratios of lung weight to body weight were greater in OSMR-KO mice than in WT mice 4 weeks after AB surgery, suggesting the exacerbation of pulmonary oedema and congestive heart failure development in OSMR-KO mice subjected to AB (Fig. 1B). The loss of OSMR robustly reduced post-AB survival. At 8 weeks after the surgery, 65% of WT control mice survived, which was comparable to the data reported by other groups [22], whereas the survival rate of OSMR-KO mice decreased to 42% (Fig. 1D). Collectively, our data illustrate the harmful effects of OSMR loss on pressure overload-induced hypertrophy.

### OSMR deficiency exacerbates cardiac dysfunction and fibrotic remodelling in pressure-overloaded hearts

**Fig. 2:**
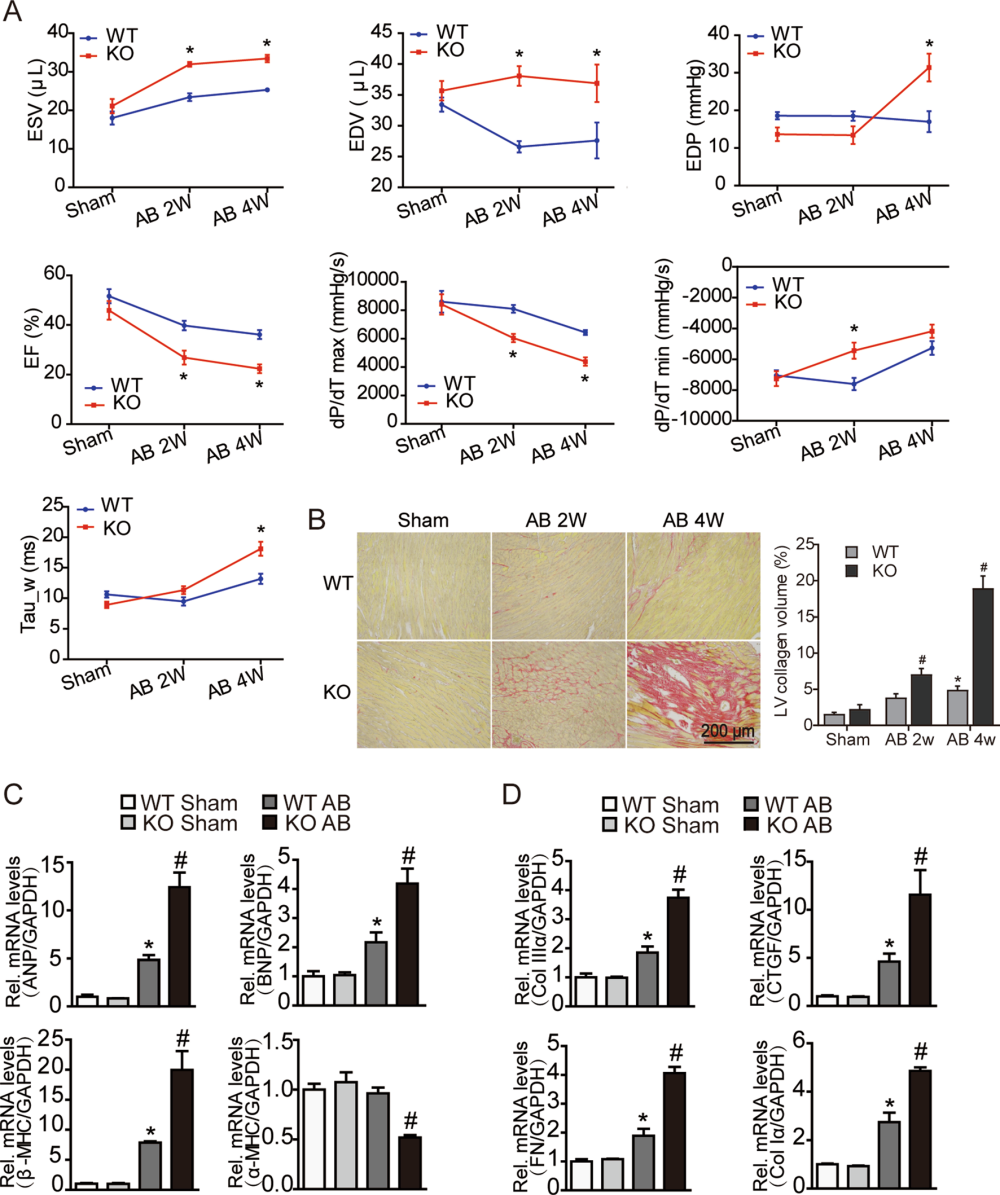
OSMR deficiency exacerbates cardiac dysfunction and fibrotic remodelling in pressure-overloaded hearts. **A** Echocardiographic parameters and haemodynamic parameters of mice with or without OSMR knockdown at the indicated times (n = 8 mice per experimental group). **B** Representative images of picrosirius red (PSR) staining of transverse heart sections in each group (n = 8 per experimental group). (C-D) RT‒PCR analysis of ANP, BNP, α-MHC and β-MHC (**C**) and Col1, Col3, Ctgf and fibronectin (FN). **D** mRNA expression in mouse hearts at 4 weeks after sham or AB surgery (n = 6 per experimental group). mRNA expression was quantified and normalized to GAPDH (fold change). Data are presented as the mean ± SEM. *P < 0.05 compared with the matched control. #P < 0.05 compared with WT/AB

We further analysed echocardiographic parameters and evaluated adverse remodelling markers at the cellular and molecular levels. As shown in Fig. 2A, echocardiographic analyses showed that the lack of the OSMR gene did not affect cardiac morphology or function under basal conditions, but after exposure to pressure overload by AB surgery, OSMR-knockout mice developed a larger left ventricular end-diastolic/systolic diameter and exhibited poorer left ventricular ejection fraction (LVEF) over time. At the tissue level, picrosirius red (PSR) staining of transverse heart sections confirmed that myocardial fibrosis was exacerbated in the hearts of OSMR knockout mice compared with WT controls (Fig. 2B). At the molecular level, AB surgery significantly upregulated the mRNA levels of the hypertrophic markers Anp, Bnp, α-Mhc, and β-Mhc and the fibrosis markers collagen Iα (Col Iα), Col IIIα, fibronectin (FN) and connective tissue growth factor (Ctgf). In contrast, these pathological changes were dramatically increased in the hearts of OSMR knockout mice compared with hearts of WT controls (Fig. 2C, D). These results suggest that OSMR deficiency promotes pathological myocardial remodelling in AB-induced hypertrophy.

### OSMR deficiency activates STAT3 signalling associated with OSM/LIFR in pressure-overloaded hearts

**Fig. 3:**
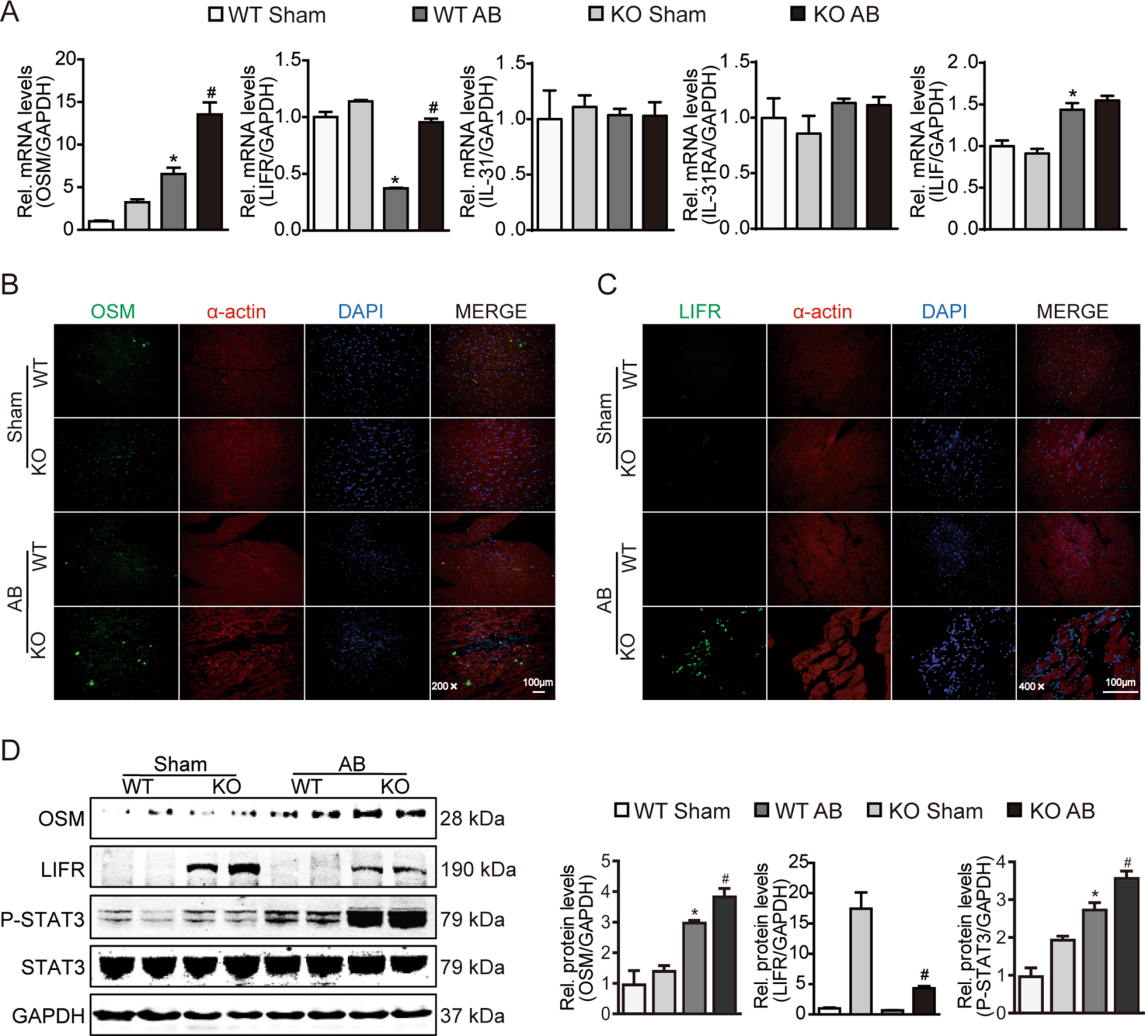
OSMR deficiency activates STAT3 signalling associated with OSM/LIFR in pressure-overloaded hearts. **A** RT‒PCR analysis of OSM, LIFR, IL-31, IL-31RA and LIF mRNA expression in mouse hearts at 4 weeks after sham or AB surgery (n = 6 per experimental group). mRNA expression was quantified and normalized to GAPDH (fold change). **B** Immunofluorescence images of OSM in mouse hearts in each group at 4 weeks after sham or AB surgery (scale bar = 100 μm for 200 × magnification). **C** Immunofluorescence images of LIFR in mouse hearts in each group at 4 weeks after sham or AB surgery (scale bar = 100 μm for 400 × magnification). **D** Representative blots and quantitative results for OSM, LIFR, phospho-STAT3 (P-STAT3) and total STAT3 protein expression in the myocardium in each group at 4 weeks after sham or AB surgery (n = 6 per experimental group). Data are presented as the mean ± SEM. *P < 0.05 compared with WT/Sham. #P < 0.05 compared with WT/AB

With the shared use of the signal transducer gp130, the biological functions of IL-6 family cytokines such as IL-6, IL-31, OSM and leukaemia inhibitory factor (LIF) largely overlap [23]. OSM and IL-31 share another common signalling receptor subunit: OSMR [24]. To explore the mechanism underlying the effect of OSMR knockout, we detected the expression of the IL-31/gp130 receptor cytokine family. Quantitative real-time polymerase chain reaction (RT‒PCR) showed that the mRNA levels of OSM and LIFR in OSMR knockout mouse myocardium were markedly upregulated in the context of pressure overload (Fig. 3A). Immunofluorescence staining of transverse heart sections demonstrated that the expression of OSM and LIFR in OSMR knockout myocardium at 4 weeks after AB surgery was higher than that in the WT group and was mainly located in the myocardial interstitium (Fig. 3B, C).

OSM has been reported to act with the receptor complex consisting of gp130 and LIFR to potentiate IL-6 signalling and further activate the following pathways: JAK/STAT, P13K/AKT, and ERK/MAPK [8]. Thus, we examined the activation state of the STAT signalling pathway. Western blotting showed that the OSM/LIFR/signal transducer and activator of transcription (STAT)3 cascade was significantly activated in the heart tissue of OSMR-KO mice after AB surgery **(**Fig. 3D**)**. These findings suggest that the hypertrophic effect of OSMR deficiency is associated with the activation of STAT3 via OSM/LIFR.

### OSMR deficiency mediates inflammation and macrophage polarization in vivo and in vitro

**Fig. 4:**
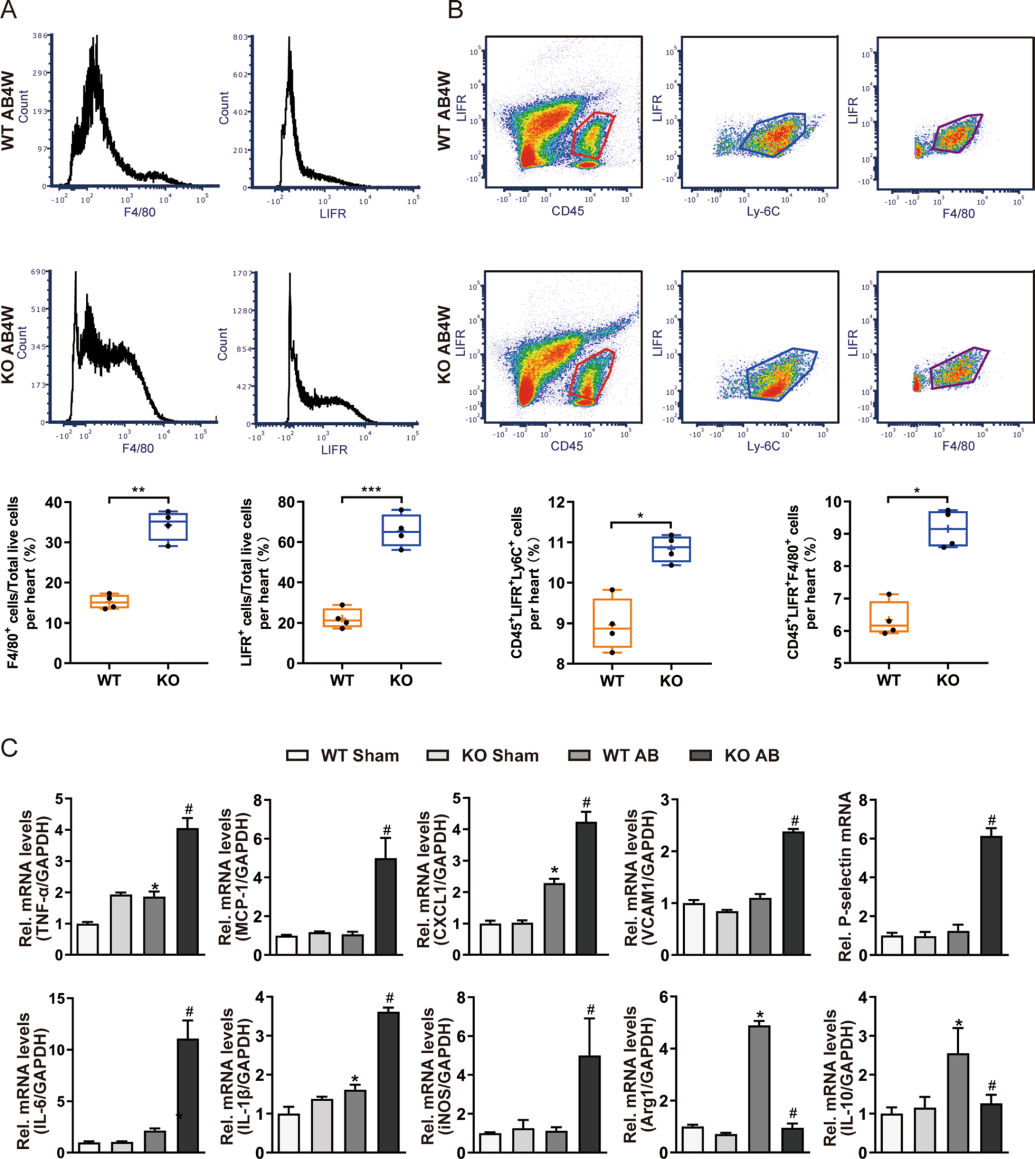
OSMR deficiency mediates inflammation and macrophage polarization in vivo. **A** Flow cytometric analysis of F4/80^+^ and LIFR^+^ cells in the heart tissue (n = 4 per experimental group). **B** Flow cytometric analysis of CD45^+^LIFR^+^Ly6C^+^ and CD45^+^LIFR^+^F4/80^+^ cells in mouse hearts at 4 weeks after sham or AB surgery (n = 4 per experimental group). **C** RT‒PCR analysis of TNF-α, MCP-1, CXCL1, VCAM1, P-selectin, IL-6, IL-1β, iNOS, Arg1 and IL-10 mRNA expression in mouse hearts at 4 weeks after sham or AB surgery (n = 6 per experimental group). mRNA expression was quantified and normalized to GAPDH (fold change). Data are presented as the mean ± SEM. *P < 0.05 compared with WT/Sham. #P < 0.05 compared with WT/AB

Evidence from previous studies demonstrated that LIFR deletion caused the upregulation of genes related to monocyte/macrophage (Mo/MΦ) inflammatory signalling in mouse embryonic ureteral tissues [25], which implied that the expression of LIFR might mediate the function and polarization of macrophages to participate in inflammatory diseases. To determine whether OSM/LIFR/STAT3 signalling was activated and transduced in macrophages, we detected the coexpression of LIFR with Mo/MΦ-specific markers and evaluated the inflammatory responses and macrophage differentiation in heart tissues. Flow cytometric (FCM) analysis showed that OSMR-KO myocardial tissues exhibited significantly increased CD45^+^LIFR^+^Ly6C^+^ and CD45^+^LIFR^+^F4/80^+^ cell infiltration compared to WT controls 4 weeks after AB surgery (Fig. 4A, B). RT‒PCR showed that the mRNA levels of inflammation markers such as TNF-α and MCP-1 and macrophage adhesion molecules such as VCAM1 and P-selectin were significantly upregulated in OSMR-KO hypertrophic hearts compared with the corresponding controls (Fig. 4C). In addition, AB-induced hypertrophic hearts lacking OSMR expressed higher levels of proinflammatory genes, including IL-6, IL-1β and iNOS, than WT controls but had significantly downregulated mRNA expression of anti-inflammatory genes, such as Arg1 and IL-10 (Fig. 4C). Collectively, these data suggest that OSMR deletion enhances inflammatory responses and limits postinjury repair following pressure overload.

**Fig. 5:**
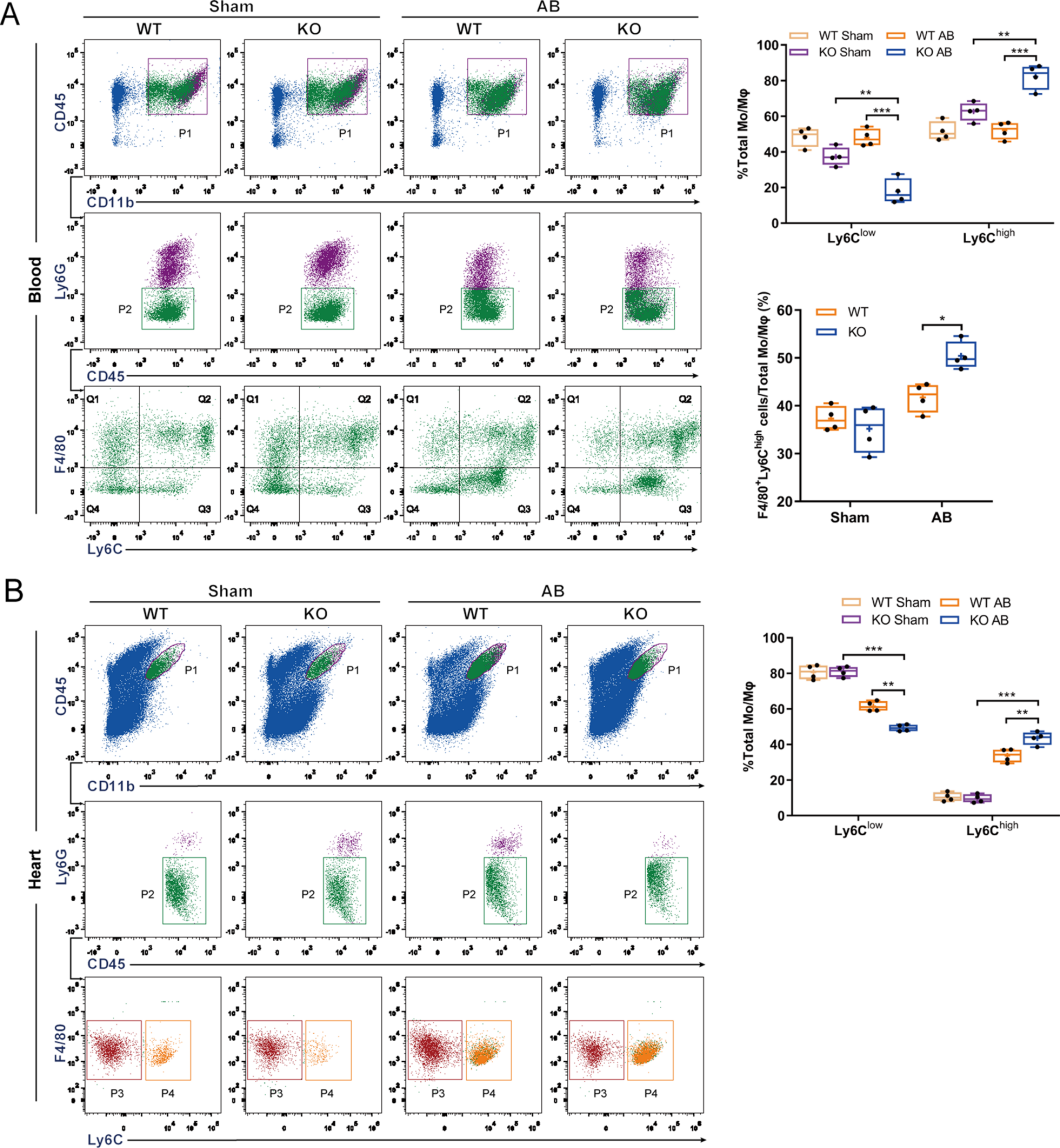
Gating and quantification of immune cells in murine hearts and blood at 4 weeks after sham or AB surgery. **A** Gating strategy for flow cytometric analysis of the leukocyte populations isolated from the blood of WT and OSMR-KO mice at 4 weeks after sham or AB surgery. Flow cytometry-based quantification of the percentage of Ly6C^high^ or Ly6C^low^ cells relative to the total monocyte/macrophage (Mo/MΦ) population and the percentage of F4/80^+^Ly6C^high^ cells relative to the total Mo/MΦ population in WT and OSMR-KO mice at 4 weeks after sham or AB surgery (n = 4 per experimental group). **B** Gating strategy for flow cytometric analysis of the leukocyte populations isolated from the hearts of WT and OSMR-KO mice at 4 weeks after sham or AB surgery. Flow cytometry-based quantification of the percentage of Ly6C^high^ or Ly6C^low^ cells relative to the total Mo/MΦ population in WT and OSMR-KO mice at 4 weeks after sham or AB surgery (n = 4 per experimental group). Data are presented as the mean ± SEM

We further characterized the macrophage population in myocardial tissue and blood of WT and OSMR-KO mice after sham or AB surgery. Accordingly, immune cells were isolated from WT and OSMR-KO blood and hearts at 4 weeks after sham or AB surgery, which were subsequently subjected to FCM to analyse the macrophage population using established gating strategies as reported in a previous study [26]. As shown in Fig. 5A, CD45^+^CD11b^+^Ly6G^−^F4/80^+^ cells were defined as the total cardiac Mo/MΦ population, and subsets of Mo/MΦ with high or low expression of Ly6C were separated and quantified. CD45^+^CD11b^+^Ly6G^−^F4/80^+^Ly6C^high^ cells are proinflammatory Mo/MΦ subtypes that mainly exert deleterious effects after the onset of pressure overload, while CD45^+^CD11b^+^Ly6G^−^F4/80^+^Ly6C^low^ cells primarily represent reparative Mo/MΦ and play a protective role in pressure-overloaded cardiac remodelling. At 4 weeks after the operation, we observed that the macrophage population in OSMR-KO mouse hearts harboured significantly higher percentages of CD45^+^CD11b^+^Ly6G^−^F4/80^+^Ly6C^high^ cells but fewer CD45^+^CD11b^+^Ly6G^−^F4/80^+^Ly6C^low^ cells (P < 0.05), which was consistent with the upregulated mRNA levels of M1 polarization markers and downregulated M2 markers (Fig. 4C). Similar results were observed in blood samples, which exhibited a significantly higher percentage of CD45^+^CD11b^+^Ly6G^−^F4/80^+^Ly6C^high^ cells in OSMR-KO mice than in WT mice (P < 0.05) (Fig. 5A, B). Overall, these flow cytometry data demonstrate that OSMR deficiency results in more abundant Ly6C^+^ proinflammatory Mo/MΦ accumulation in the heart and blood in the context of pressure overload.

**Fig. 6:**
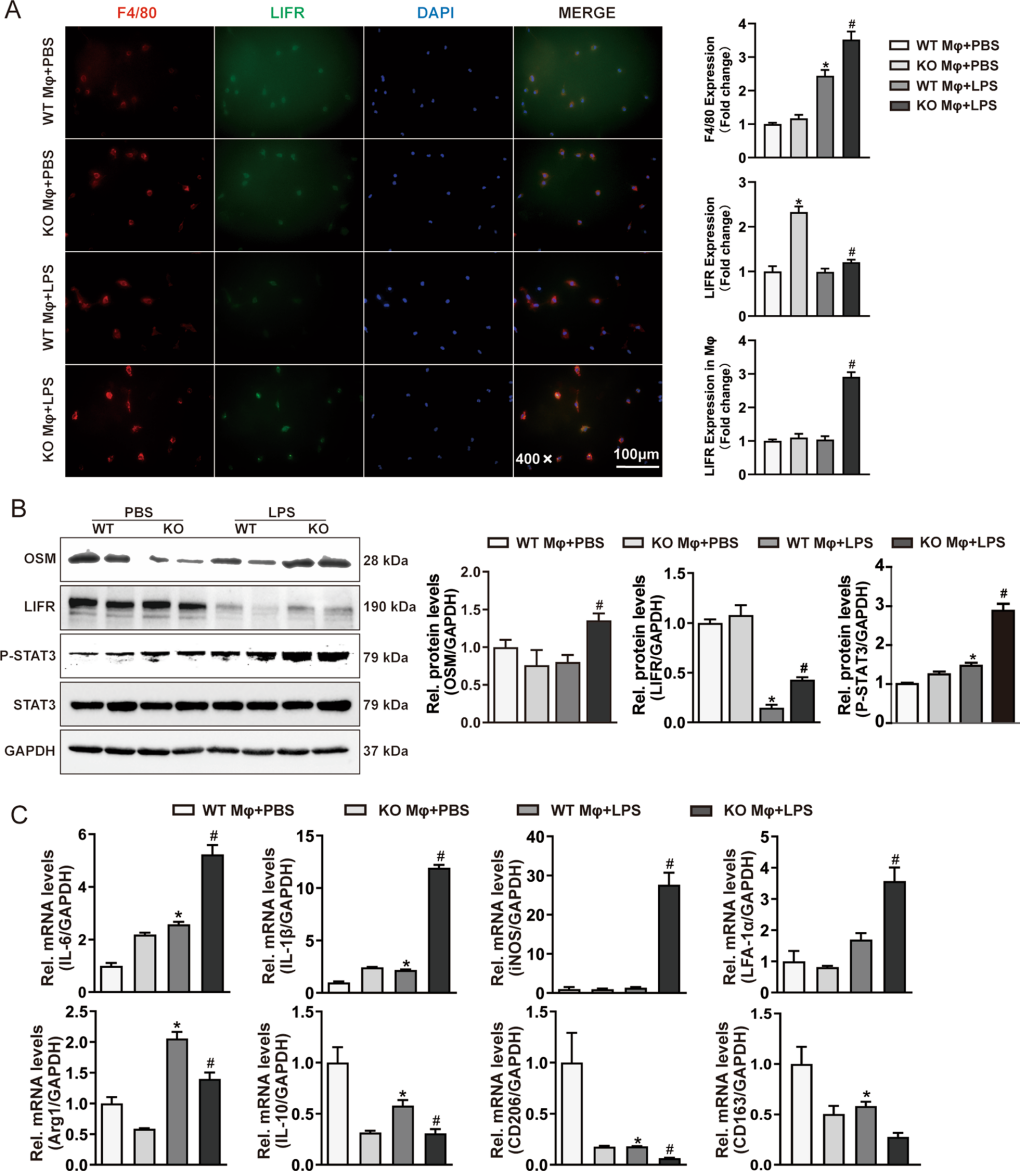
OSMR deficiency mediates inflammation and macrophage polarization in vitro. **A** Immunofluorescence images of F4/80 (red) and LIFR (green) in WT and KO bone marrow-derived macrophages (BMDMs) treated with LPS (scale bar = 100 μm for 400 × magnification. **B** Representative blots and quantitative results for OSM, LIFR, phospho-STAT3 (P-STAT3) and total STAT3 protein expression in BMDMs treated with LPS (n = 6 per experimental group). **C** RT‒PCR analysis of M1-like (IL-6, IL-1β, iNOS, LFA-1α) and M2-like (Arg1, IL-10, CD206, CD163) macrophage marker mRNA expression in BMDMs treated with LPS (n = 6 per experimental group). mRNA expression was quantified and normalized to GAPDH (fold change). Data are presented as the mean ± SEM. *P < 0.05 compared with WT MΦ/PBS. #P < 0.05 compared with WT MΦ/LPS

For in vitro experiments, BMDMs were isolated from WT and OSMR-KO mice and cultured. Double immunofluorescence staining showed that the expression of LIFR was increased in OSMR-KO macrophages stimulated with LPS compared with MΦs from WT mice (Fig. 6A). Concomitantly, we observed a similar pattern in OSM/LIFR/STAT3 signalling activation in OSMR-KO BMDMs treated with LPS (Fig. 6B). In addition, RT-PCR analysis showed that OSMR deletion increased the mRNA levels of M1 macrophage markers (IL-6, IL-1β, iNOS, LFA-1α) while decreasing the expression of M2 macrophage markers (Arg1, IL-10, CD206, CD163) after LPS stimulation (Fig. 6C). These data suggest that the loss of OSMR promotes the development of cardiac hypertrophy, at least partly, by modulating macrophage polarization and activating the OSM/LIFR/STAT3 signal transduction pathway in macrophages.

### Adoptive transfer of Osmr-KO macrophages into WT mice aggravates pressure overload-induced cardiac remodelling and cardiac dysfunction

**Fig. 7:**
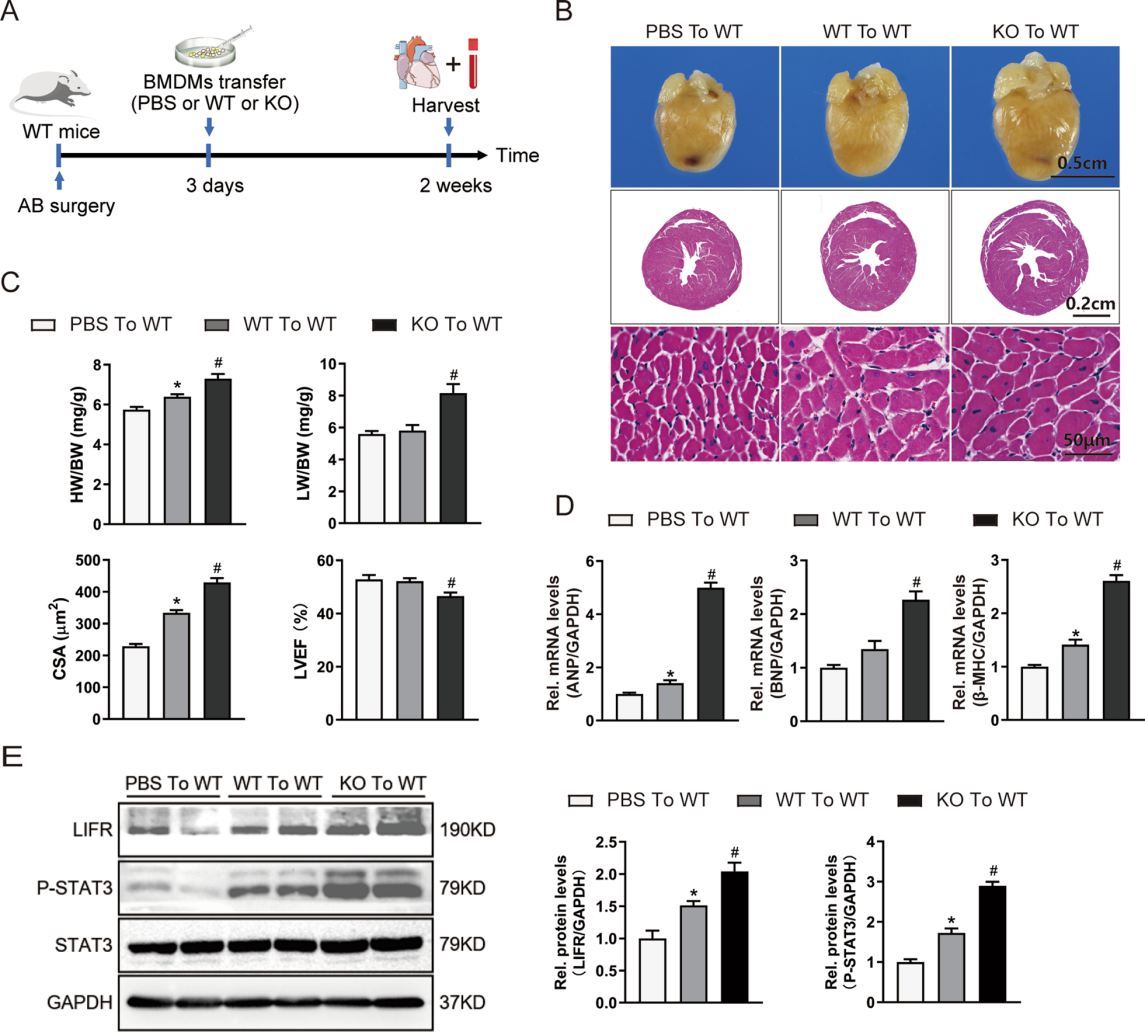
Adoptive transfer of OSMR-KO macrophages into WT mice aggravates pressure overload-induced cardiac remodelling and cardiac dysfunction. **A** Schematic diagram outlining the adoptive transfer experiment. PBS or BMDMs were obtained from WT or OSMR-KO mice and adoptively transferred via the tail vein into WT mice 3 days after AB surgery. **B** Representative gross morphology and haematoxylin and eosin-stained heart sections of mice subjected to AB surgery and adoptive transfer (n = 8 mice per experimental group). **C** Heart weight to body weight (HW/BW) ratios, lung weight to body weight (LW/BW) ratios, cross-sectional areas and echocardiographic parameters (LVEF) of mice subjected to AB surgery and adoptive transfer (n = 8 mice/100 + cells per experimental group). **D** RT‒PCR analysis of ANP, BNP, and β-MHC mRNA expression in murine hearts (n = 6 per experimental group). mRNA expression was quantified and normalized to GAPDH (fold change). **E** Representative blots and quantitative results for OSM, LIFR, phospho-STAT3 (P-STAT3) and total STAT3 protein expression in murine hearts (n = 6 per experimental group). Data are presented as the mean ± SEM. *P < 0.05 compared with PBS to WT. #P < 0.05 compared with WT to WT

To further confirm the relationship between macrophage function and cardiac hypertrophy in OSMR-KO mice, we isolated BMDMs from WT and OSMR-KO mice, and the cells were subsequently used in an adoptive transfer experiment. Specifically, WT or OSMR-KO BMDMs were transferred into WT mice 3 days after the AB operation (Fig. 7A). At 2 weeks after the onset of pressure overload, OSMR-KO BMDM transfer significantly aggravated the AB-induced elevation of HW/BW and LW/BW ratios and the upregulation of ANP, BNP and β-MHC mRNA levels compared to PBS or WT BMDM transfer, although there were no significant differences in cardiac function, as indicated by the echocardiographic measurement of LVEF **(**Fig. 7B-D**)**. Furthermore, western blotting demonstrated that OSMR-KO BMDM transfer activated LIFR/STAT3 signalling (Fig. 7E).

**Fig. 8:**
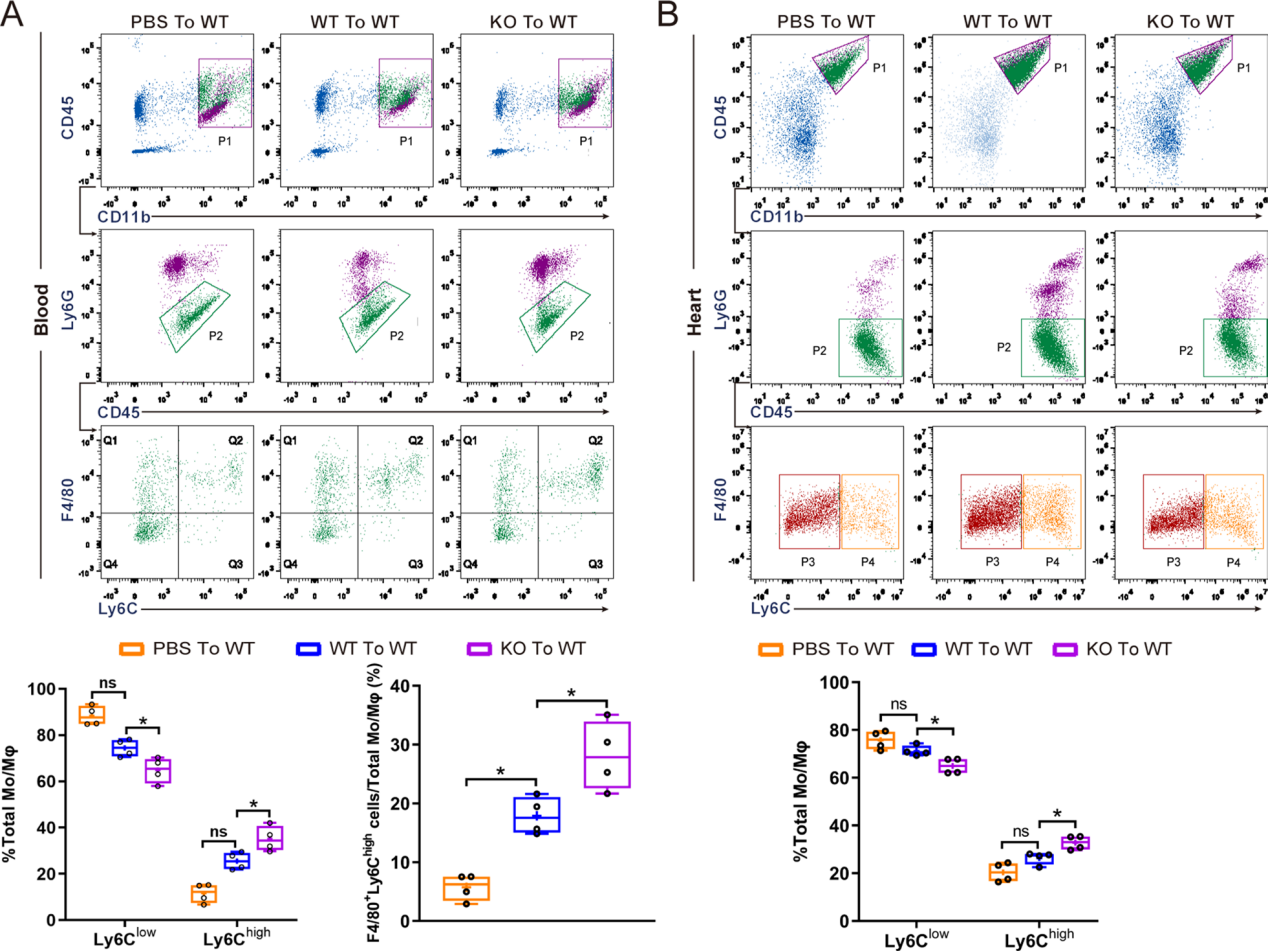
Gating and quantification of immune cells in the heart and blood of mice that underwent adoptive transfer. **A** Gating strategy for flow cytometric analysis of the leukocyte population isolated from the blood of WT mice that underwent adoptive transfer of PBS, WT and OSMR-KO BMDMs after AB surgery. Flow cytometry-based quantification of the percentage of Ly6C^high^ or Ly6C^low^ cells relative to the total monocyte/macrophage (Mo/MΦ) population and the percentage of F4/80^+^Ly6C^high^ cells relative to the total Mo/MΦ population. (n = 4 per experimental group). **B** Gating strategy for flow cytometric analysis of the leukocyte population isolated from the hearts of WT mice that underwent adoptive transfer of PBS, WT and OSMR-KO BMDMs after AB surgery. Flow cytometry-based quantification of the percentage of Ly6C^high^ or Ly6C^low^ cells relative to the total Mo/MΦ population. (n = 4 per experimental group). Data are presented as the mean ± SEM

In addition, flow cytometry revealed that OSMR-KO BMDM transfer increased the percentages of CD45^+^CD11b^+^Ly6G^−^F4/80^+^Ly6C^high^ cells but decreased those of CD45^+^CD11b^+^Ly6G^−^F4/80^+^Ly6C^low^ cells (P < 0.05) in heart tissue and blood compared with PBS or WT BMDM transfer (Fig. 8A, B).

### LIFR knockdown mitigates the hypertrophic effect of OSMR deficiency after AB surgery

**Fig. 9:**
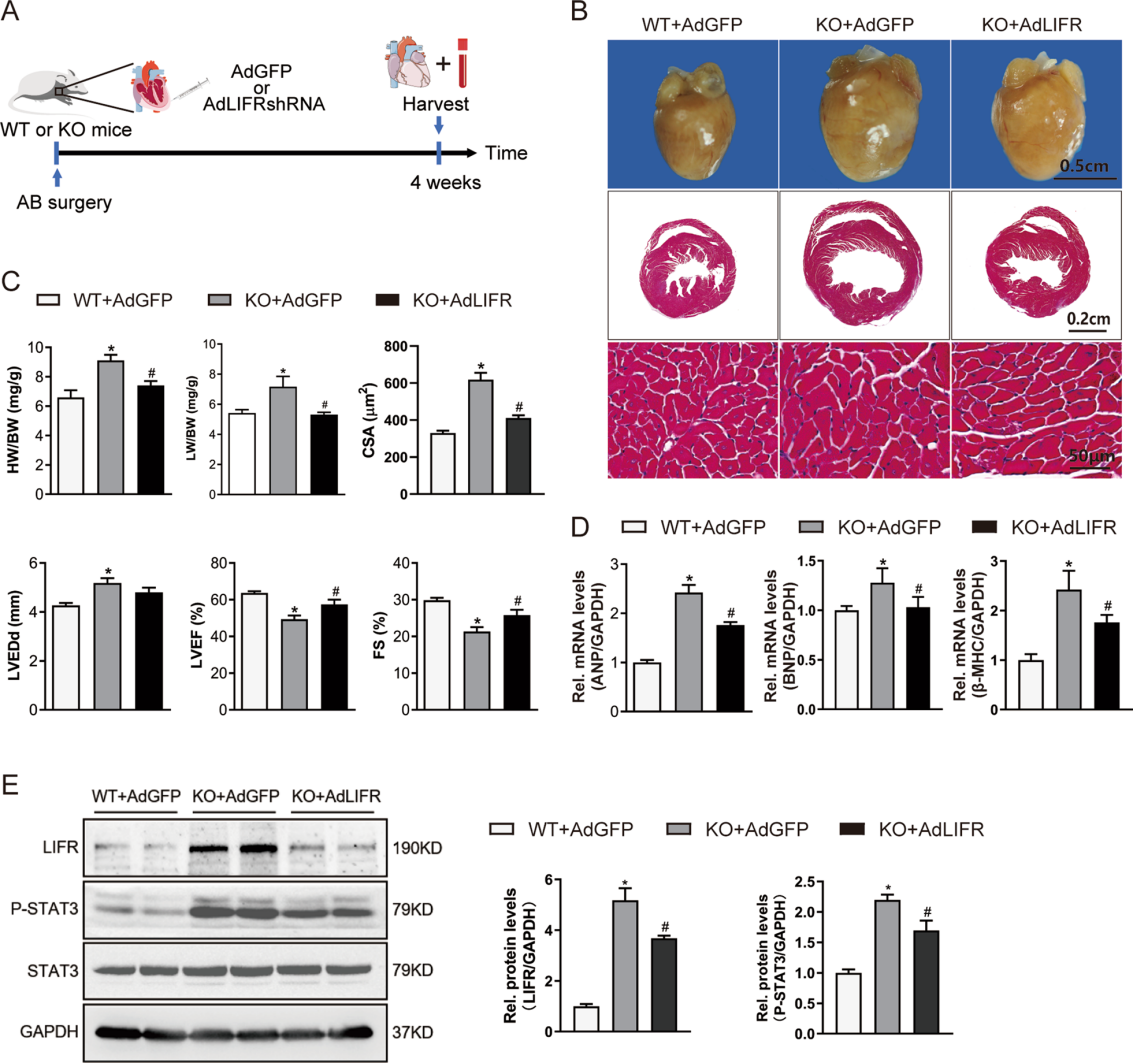
LIFR knockdown mitigates the hypertrophic effect of OSMR deficiency after AB surgery. **A** Schematic diagram outlining the intramyocardial injection experiment. Adenovirus-mediated short hairpin RNA (shRNA) transfer was performed through direct intramyocardial injection in the left ventricular free wall. **B** Representative gross morphology and haematoxylin and eosin-stained heart sections of mice subjected to AB surgery (n = 8 mice per experimental group). **C** Heart weight to body weight (HW/BW) ratios, lung weight to body weight (LW/BW) ratios, cross-sectional areas and echocardiographic parameters (LVEDd, LVEF, FS) of mice subjected to AB surgery (n = 8 mice/100 + cells per experimental group). **D** RT‒PCR analysis of ANP, BNP, and β-MHC mRNA expression in murine hearts (n = 6 per experimental group). mRNA expression was quantified and normalized to GAPDH (fold change). **E** Representative blots and quantitative results for OSM, LIFR, phospho-STAT3 (P-STAT3) and total STAT3 protein expression in murine hearts (n = 6 per experimental group). Data are presented as the mean ± SEM. *P < 0.05 compared with WT + AdGFP. #P < 0.05 compared with KO + AdGFP

**Fig. 10:**
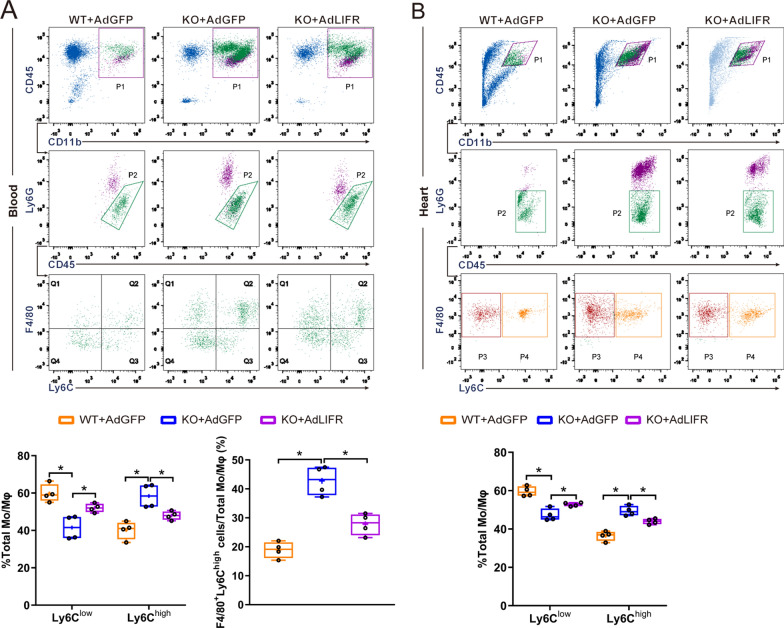
Gating and quantification of immune cells in the heart and blood of mice that received intramyocardial transfer of adenovirus. **A** Gating strategy for flow cytometric analysis of the leukocyte population isolated from the blood of WT and OSMR-KO mice that received intramyocardial transfer of AdGFP and AdLIFRshRNA after AB surgery. Flow cytometry-based quantification of the percentage of Ly6C^high^ or Ly6C^low^ cells relative to the total monocyte/macrophage (Mo/MΦ) population and the percentage of F4/80^+^Ly6C^high^ cells relative to the total Mo/MΦ population. (n = 4 per experimental group). **B** Gating strategy for flow cytometric analysis of the leukocyte population isolated from the hearts of WT and OSMR-KO mice that received intramyocardial transfer of AdGFP and AdLIFRshRNA after AB surgery. Flow cytometry-based quantification of the percentage of Ly6C^high^ or Ly6C^low^ cells relative to the total Mo/MΦ population. (n = 4 per experimental group). Data are presented as the mean ± SEM

We determined whether OSMR deficiency lost its hypertrophic effect when LIFR/STAT3 signalling was blocked. We used mice with cardiac-specific knockdown of LIFR induced by intramyocardial injection of LIFR-shRNA (Fig. 9A). As expected, LIFR blockade was accompanied by STAT3 signalling interruption in the hearts of OSMR-KO mice + LIFR-shRNA, which was confirmed by western blot analysis (Fig. 9E). We observed that OSMR-KO mice + LIFR-shRNA exhibited an attenuated hypertrophic phenotype compared with OSMR-KO mice + GFP in response to pressure overload, which was reflected by the HW/BW ratio, cross-sectional area and echocardiographic alterations (Fig. 9B–D). Moreover, we found that the percentage of CD45^+^CD11b^+^Ly6G^−^F4/80^+^Ly6C^high^ cells was decreased, while the percentage of CD45^+^CD11b^+^Ly6G^−^F4/80^+^Ly6C^low^ cells was significantly increased in the heart and blood of OSMR-KO mice + LIFR-shRNA compared with that of WT mice + GFP or OSMR-KO mice + GFP (P < 0.05) (Fig. 10A, B). Taken together, these data suggest that LIFR/STAT3 signalling blockade reversed adverse pressure overload-induced cardiac remodelling in the context of OSMR deficiency and ultimately substantiated a crucial role for LIFR in regulating the inflammatory phenotype of macrophages.

**Fig. 11:**
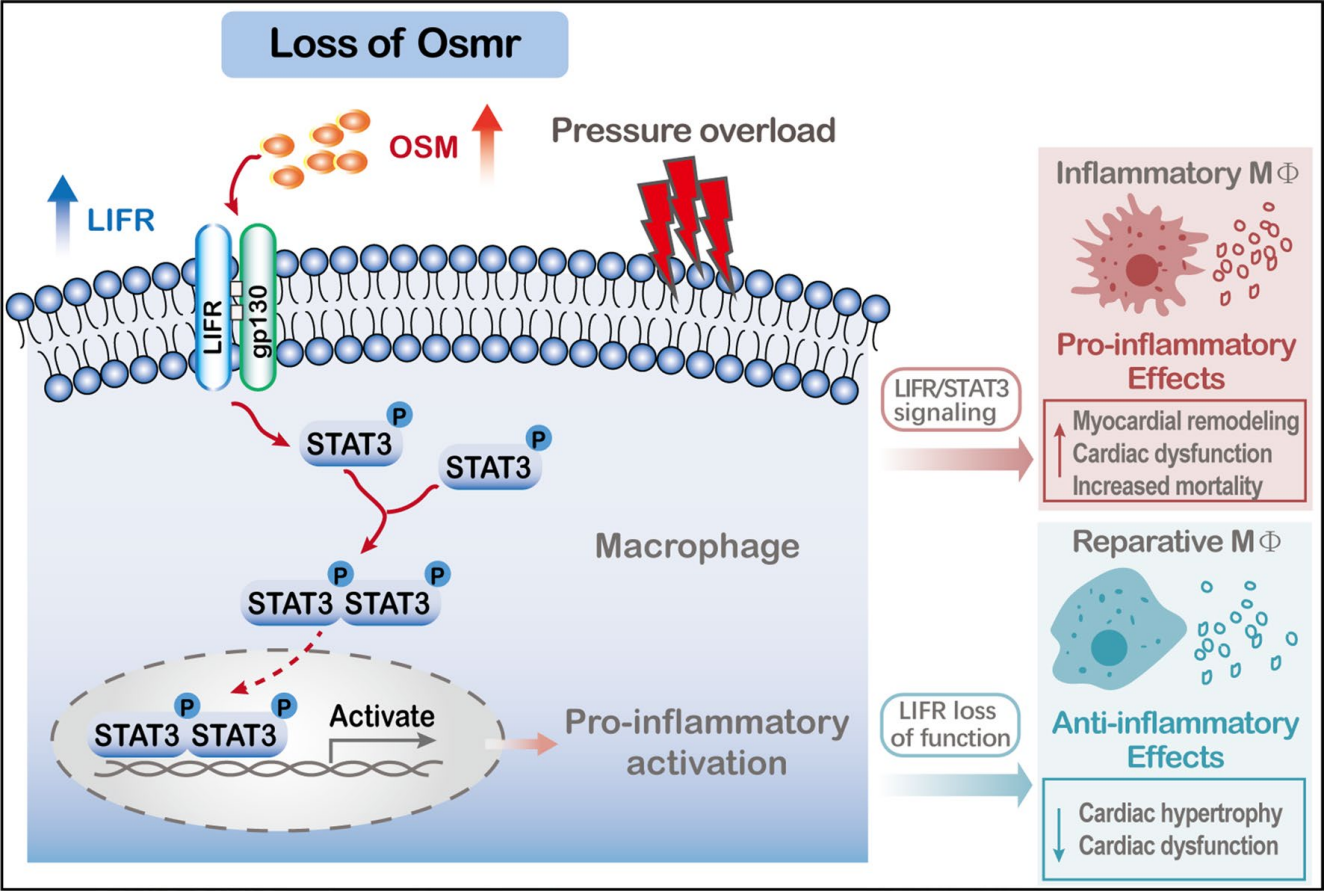
Schematic images of the regulatory mechanisms of Osmr deficiency on pathological cardiac hypertrophy

## Discussion

Previous studies demonstrated that therapeutic application of recombinant mOSM markedly improved cardiac performance and dramatically increased survival after infarction, whereas strains with OSMR deletion showed a reduced ejection fraction and high mortality [12, 27, 28]. In this study, we disclosed a previously unrecognized role of OSMR in the context of cardiac remodelling induced by chronic pressure overload. First, using different approaches, we observed that the expression of OSMR changed significantly in murine hypertrophic hearts induced by pressure overload, as well as in BMDMs treated with Ang II. By performing loss-of-function animal experiments, we uncovered the detrimental effects of OSMR deficiency on the pathogenesis of cardiac hypertrophy. Knockout of OSMR promoted adverse pressure overload-induced cardiac hypertrophy and cardiac dysfunction. Mechanistically, OSMR deletion in hypertrophic models led to the activation of OSM/LIFR/STAT3 signalling and promoted a proresolving macrophage phenotype that exacerbated inflammation and impaired cardiac repair during remodelling. In addition, adaptive transfer of OSMR-knockout BMDMs accentuated cardiac remodelling, while myocardium-specific disruption of LIFR impaired the hypertrophic effect of OSMR deficiency and decreased the phosphorylation of STAT3 signalling. Based on the in vivo and in vitro data, we concluded that OSMR deficiency could contribute to the development of pressure overload-induced cardiac hypertrophy by modulating macrophage function and the OSM/LIFR/STAT3 signalling pathway (see Fig. 11).

Gp130 is known as the common receptor subunit and signal transducer of IL-6 cytokines. The binding of a ligand to its receptor induces homodimerization of gp130 (upon binding of IL-6 and IL-11) or heterodimerization of gp130 with LIFR (following binding of LIF, OSM, CT-1 and CNTF) or OSMR (for OSM) [23]. It was first acknowledged that murine OSM (mOSM) bound first to gp130 and then formed a high-affinity complex only with OSMR [29]. However, subsequent studies have shown that in the absence of OSMR in mice, mOSM acts through LIFR to phosphorylate STAT3 [30, 31]. Humanoid OSM mutated proteins were also reported to act through both OSMR and LIFR receptors to protect against ischaemia-induced myocardial injury [32]. In this study, we found that LIFR mRNA was expressed in adult murine hearts; however, the LIFR protein was not expressed in adult WT murine hearts. Suppressor of cytokine signalling 3 (SOCS3) was thought to suppress LIFR signalling in mouse trophoblasts [33]. Interestingly, we observed that knockout of OSMR augmented the protein expression of LIFR, which was reduced after AB surgery. We further observed the upregulation of OSM and increased phosphorylation of STAT3, which indicated that the OSM/LIFR/STAT3 signalling axis is involved in the hypertrophic response during OSMR deficiency. In addition, these novel findings suggested a causal link between LIFR and cardiac hypertrophy rather than a simple compensatory response. Whether this effect is independent of OSMR requires further elucidation.

As a key hallmark, inflammation contributes to the development of pressure-overloaded cardiac remodelling, in which monocytes/macrophages play an important role [34, 35]. Due to great heterogeneity and plasticity in their phenotype and function, macrophages are the main immune cells that are altered during the 2–5 week onset of pressure overload [36]. During this stage, cardiac macrophages transform into proinflammatory subtypes, releasing abundant cytokines, including IL-6, TNF-α and IFN-γ, which are responsible for the progression of remodelling and deterioration of cardiac function [37].

Many studies have determined the association between IL-6 family cytokines and inflammatory diseases, and more importantly, OSM is released by monocytes/macrophages, dendritic cells and T lymphocytes in inflammatory conditions, and upon binding to their respective receptor complexes, these factors signal mainly via the JAK/STAT pathway [24]. OSM is secreted by macrophages and can act on GP130/OSMR or GP130/LIFR receptors on glioblasts and activate STAT3 in these cells [38]. Stimulation of the macrophage cell line RAW264.7 with OSMR results increases the expression of M2 markers (IL-10, Arg1, and CD206), and the loss of OSMR leads to the polarization of adipose tissue macrophages to M1 phenotypes that increase inflammation in a mouse model of metabolic diseases [15]. These studies show a close relationship between OSM/OSMR associated with downstream signalling, inflammation and macrophages. Moreover, sustained activation of the OSMR/gp130 cascade is associated with macrophage infiltration and the development of heart failure in adult patients with various chronic heart diseases. Inhibition of Ly6C^high^ monocyte-derived macrophage infiltration into the myocardium in the early phase could significantly delay the progression to HF [37, 39]. In the present study, we observed that the loss of OSMR led to increased numbers of Ly6C^high^ proinflammatory Mo/MΦ but fewer reparative Ly6C^low^ Mo/MΦ in the heart and blood, along with the upregulation of inflammatory factors, at 4 weeks after AB surgery. Interestingly, a previous study indicated that genetic inactivation of OSMR reduced the accumulation of macrophages (Ly6C^high^ and Ly6C^low^ macrophages) in the ischaemic myocardium in mice [27], which seemed to be contrary to our current findings. This paradoxical result might be attributed to the different disease models and the alternative activation of LIFR/STAT3 signalling in the hypertensive heart.

It has been well established that STAT3-dependent downstream pathways play critical roles in pathological hypertrophy. Phosphorylated STAT3 forms homo/heterodimers and translocates from the cytoplasm into the nucleus, through which the transcription of downstream genes related to cardiac hypertrophy is activated [23, 40]. Previous studies have suggested that STAT3 signalling functions as a key downstream regulator of OSM to mediate various cellular processes in different cell types, and more importantly, STAT3 and its related signalling axis can indirectly affect the pathophysiological process of cardiovascular events by regulating inflammation or macrophages [41, 42]. Moreover, studies have illustrated that STAT3 signalling is involved in macrophage activation and polarization, blood cell formation and immunity [42, 43]. By performing immunofluorescence staining, we found that OSM and LIFR were mainly located in the myocardial interstitium of hypertrophic hearts. We thus asked whether OSM/LIFR/STAT3 signalling was activated in macrophages and whether it participated in OSMR deletion-regulated cardiac hypertrophy.

In the present study, we observed that the coexpression of LIFR and F4/80 was augmented in an in vivo mouse AB model and cultured BMDMs with OSMR knockout, which was accompanied by the upregulation of OSM and the phosphorylation of STAT3, indicating the involvement of macrophage-associated STAT3 in OSMR deletion-regulated cardiac hypertrophy. We further confirmed that the LIFR/STAT3 axis in macrophages modulated the hypertrophic response after the adaptive transfer of BMDMs. These observations suggested that the OSM/LIFR/STAT3 signalling regulatory axis in macrophages might be an underlying mechanism in OSMR deletion-regulated cardiac hypertrophy. Furthermore, our findings were supported by experiments on cardiac-specific knockdown of LIFR via intramyocardial injection of Ad-LIFR-shRNA, and the data altogether indicated the importance of addressing whether and how LIFR exerts additional effects on the phenotype and function of macrophages and the development of cardiac remodelling in the future.

This study has several limitations. First, global knockout mice were used in our study instead of macrophage-specific knockout mice. Second, although we have demonstrated the detrimental role of OSMR deficiency in pressure overload-induced hypertrophy, the effects of OSMR overexpression on macrophages and AB-induced cardiac remodelling still require further clarification.

In summary, the present study shows for the first time that OSMR deficiency exerts adverse effects on the development of pressure overload-induced cardiac hypertrophy, which is mediated by OSM/LIFR/STAT3 signalling associated with macrophages and inflammation. Our data provide new insights into the role of OSMR in diseases and confirm the association between OSMR and macrophage function in cardiac remodelling, thus providing a new opportunity to develop novel treatment strategies for adverse cardiac remodelling by targeting macrophages.

## Supplementary Information

Additional file 1: Tables S1. Primary antibodies used in our study. Tables S2. The primers used for RT‒PCR in our study. Figure S1. OSMR expression in hypertrophic mouse hearts and different cells. Figure S2. Genotyping of OSMR gene knockoutmice.

## Data Availability

All data generated in this study are available on reasonable request from the corresponding author.

## Abbreviations

AB: Aortic banding
α-MHC: α-Myosin heavy chain
ANP: Atrial natriuretic peptide
Arg1: Arginase 1
β-MHC: β-Myosin heavy chain
BMDMs: Bone marrow-derived macrophages
BNP: β-Type natriuretic peptide
BW: Body weight
CSA: Cross-sectional area
CT-1: Cardiotrophin-1
CTGF: Connective tissue growth factor
CNTF: Ciliary neurotrophic factor
FN: Fibronectin
GP130: Glycoprotein 130
HF: Heart failure
HW: Heart weight
IL: Interleukin
iNOS: Inducible nitric oxide synthase
KO: Knockout
LFA-1α: Leukocyte function-associated molecule 1α
LIFR: Leukaemia inhibitory factor receptor
LPS: Lipopolysaccharide
MCP-1: Monocyte chemotactic protein 1
M-CSF: Recombinant mouse macrophage colony-stimulating factor
Mo/MΦ: Monocytes/macrophages
OSM(R): Oncostatin M (receptor beta)
shRNA: Short hairpin RNA
STAT3: Signal transducer and activator of transcription 3
TNF: Tumour necrosis factor
TL: Tibia length
VCAM1: Vascular cell adhesion molecule 1
WT: Wild type
