# Supplementary material for: OSMR deficiency aggravates pressure overload-induced cardiac hypertrophy by modulating macrophages and OSM/LIFR/STAT3 signalling
Source: J Transl Med. 2023 Apr 29;21:290. doi: 10.1186/s12967-023-04163-x (PMC10149029; doi:10.1186/s12967-023-04163-x)
Supplement: Supplementary file 1 — Additional file 1: Tables S1. Primary antibodies used in our study. Tables S2. The primers used for RT‒PCR in our study. Figure S1. OSMR expression in hypertrophic mouse hearts and different cells. Figure S2. Genotyping of OSMR gene knockoutmice. [file 12967_2023_4163_MOESM1_ESM.docx]

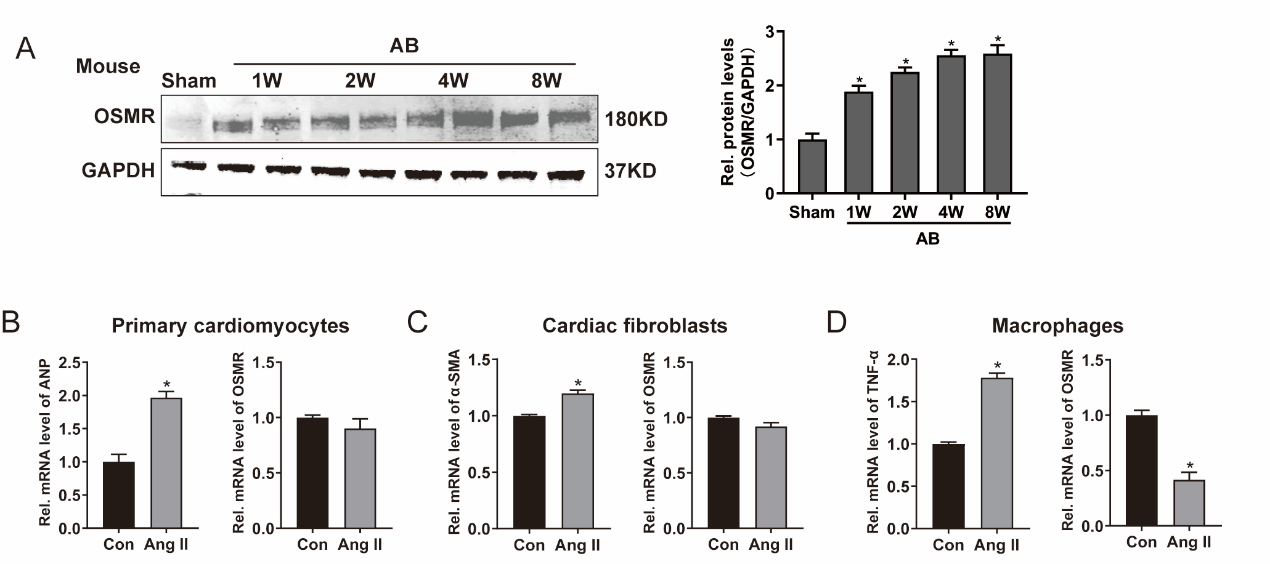


**Figure S1.** **The OSMR expression in mouse hypertrophic heart and different cells.**

(A) Representative Western blot and quantitative results of OSMR and GAPDH in mouse hypertrophic heart (n=6). Protein expression was normalized to GAPDH before quantitative analysis. (B-D) RT-PCR analysis of OSMR mRNA expression in primary cardiomyocytes, cardiac fibroblasts and bone marrow-derived macrophages (n=6 per experimental group). mRNA expression was quantified and normalized to GAPDH (fold change). Data are presented as mean ± SEM. *p < 0.05 compared with the matched control.


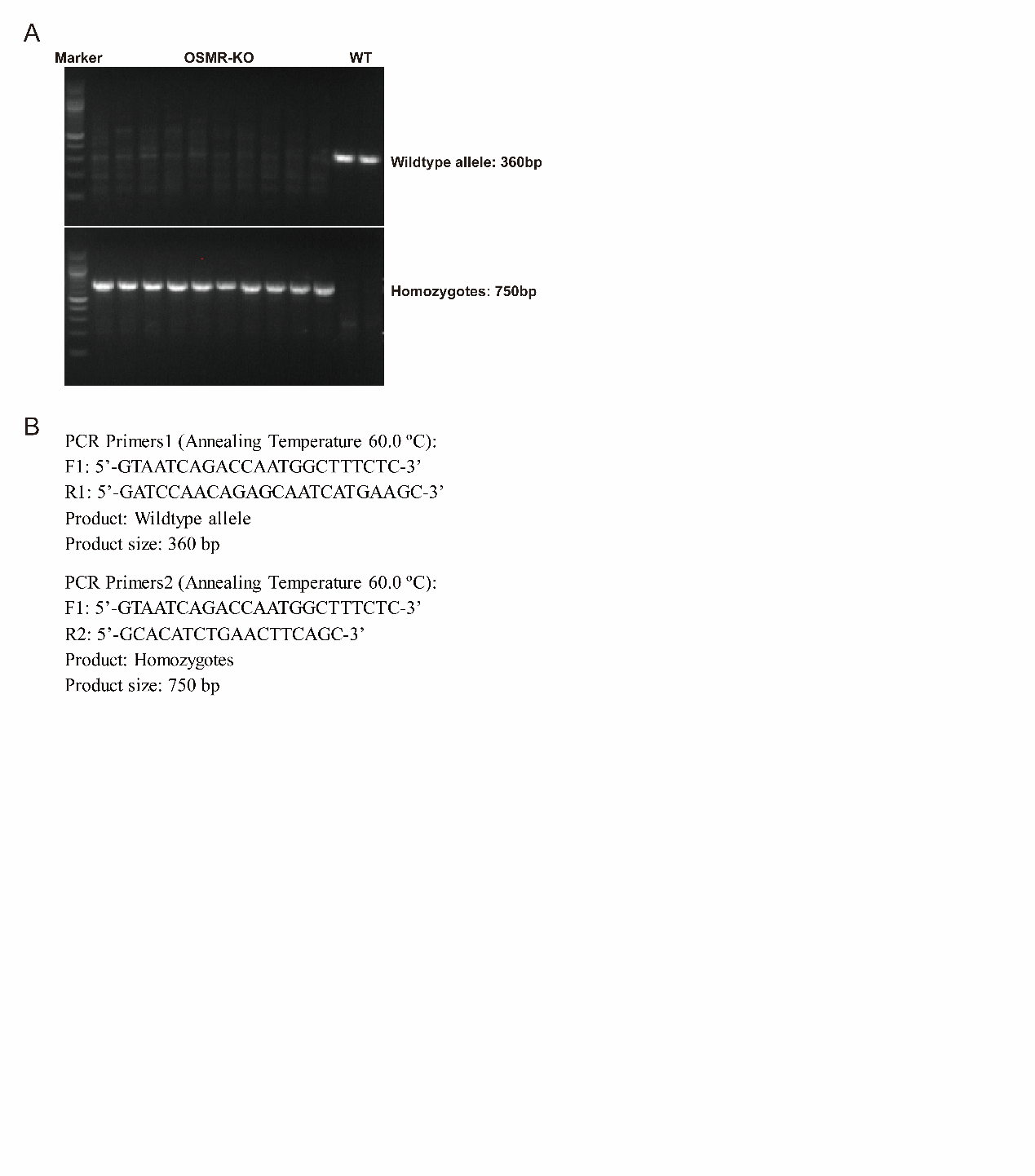


**Figure S2.** **Genotyping of OSMR gene knockout (OSMR-KO) Mice.**

(A) Agarose gel electrophoresis for OMSR-KO homozygous and WT Mice. (B) PCR Primers for genotypic identification.

**Table S1.** **Primary antibodies used in our study.**

| Antibodies | Vendor or Source | Catalog # | Working concentration |
| --- | --- | --- | --- |
| Anti-OSM | Santa Cruz | sc-374039 | WB (1:100)  IF (1:50) |
| Anti-OSMR beta  (mouse) | Santa Cruz | sc-376511 | WB (1:100) |
| Anti-OSMR beta  (human) | Santa Cruz | sc-9992 | WB (1:100) |
| Anti-LIFR | Abcam | ab101228 | WB (1:1000) |
| Anti-STAT3 | Cell Signaling Technology | 30835S | WB (1:1000) |
| Anti-phospho-STAT3 (Tyr705) | Cell Signaling Technology | 9145 | WB (1:1000) |
| Anti-GAPDH | Cell Signaling Technology | 2118 | WB (1:1000) |
| Anti-F4/80 | Abcam | ab6640 | IF (1:50) |
| Anti-LIFR | Proteintech | 22779-1-AP | IF (1:50) |
| Anti-CD16/32 (2.4G2) | BioLegend | 101302 | FC (1.0 µg per 10^6^ cells in 100 µL volume) |
| Anti-CD45-APC/ Cy7 (30-F11) | BioLegend | 103116 | FC (0.25 µg per 10^6^ cells in 100 µl volume) |
| Anti-CD11b-FITC (M1/70) | BioLegend | 101206 | FC (0.25 µg per 10^6^ cells in 100 µl volume) |
| Anti-Ly-6G- APC (1A8) | BioLegend | 127614 | FC (0.06 µg per 10^6^ cells in 100 µl volume) |
| Anti-F4/80-PE  (BM8) | BioLegend | 123110 | FC (1.0 µg per 10^6^ cells in 100 µl volume) |
| Anti-Ly-6C-PE/Cy7 (HK1.4) | BioLegend | 128018 | FC (0.06 µg per 10^6^ cells in 100 µl volume) |

**Table S2.** **The primers used for RT-PCR in our study.**

| Gene | Species |  | Sequence (5’-3’) |
| --- | --- | --- | --- |
| *α-Mhc* | Mouse | Forward | GTCCAAGTTCCGCAAGGT |
|  |  | Reverse | AGGGTCTGCTGGAGAGGTTA |
| *Arg1* | Mouse | Forward | CTCCAAGCCAAAGTCCTTAGAG |
|  |  | Reverse | AGGAGCTGTCATTAGGGACATC |
| *Anp* | Mouse | Forward | ACCTGCTAGACCACCTGGAG |
|  |  | Reverse | CCTTGGCTGTTATCTTCGGTACCGG |
| *β-Mhc* | Mouse | Forward | CCGAGTCCCAGGTCAACAA |
|  |  | Reverse | CTTCACGGGCACCCTTGGA |
| *Bnp* | Mouse | Forward | GAGGTCACTCCTATCCTCTGG |
|  |  | Reverse | GCCATTTCCTCCGACTTTTCTC |
| *Cd206* | Mouse | Forward | AAACACAGACTGACCCTTCCC |
|  |  | Reverse | GTTAGTGTACCGCACCCTCC |
| *Collagen Ia* | Mouse | Forward | GCTTCAGTGGTTTGGATGGT |
|  |  | Reverse | AGGGCGACCTCTCTCACC |
| *Collagen IIIa* | Mouse | Forward | CGTAAGCACTGGTGGACAGA |
|  |  | Reverse | AGCTGCACATCAACGACATC |
| *Ctgf* | Mouse | Forward | CAAAGCAGCTGCAAATACCA |
|  |  | Reverse | GGCCAAATGTGTCTTCCAGT |
| *Fibronectin* | Mouse | Forward | CACGGAGGCCACCATTACT |
|  |  | Reverse | CTTCAGGGCAATGACGTAGAT |
| *Gapdh* | Mouse | Forward | ACTCCACTCACGGCAAATTC |
|  |  | Reverse | TCTCCATGGTGGTGAAGACA |
| *iNos* | Mouse | Forward | GGGAATCTTGGAGCGAGTTG |
|  |  | Reverse | GTGAGGGCTTGGCTGAGTGA |
| *Il-1β* | Mouse | Forward | GGCAGGCAGTATCACTCATT |
|  |  | Reverse | AAGGTGCTCATGTCCTCATC |
| *Il-31* | Mouse | Forward | TCAGCAGACGAATCAATACAGC |
|  |  | Reverse | TCGCTCAACACTTTG ACTTTCT |
| *Il-31ra* | Mouse | Forward | AGACAGGAAGTTGCGATTGCT |
|  |  | Reverse | TGTATGTGGTAGCCAAATGTTTTCTC |
| *Il-6* | Mouse | Forward | CCGGAGAGGAGACTTCACAG |
|  |  | Reverse | TCCACGATTTCCCAGAGAAC |
| *Il-10* | Mouse | Forward | GCCAAGCCTTATCGGAAATG |
|  |  | Reverse | GGGAATTCAAATGCTCCTTGAT |
| *Lfa-1α* | Mouse | Forward | CATGCAGCCTATCCTGAGAC |
|  |  | Reverse | TAATCGCACCCAGTAGGCATC |
| *Lif* | Mouse | Forward | CCCATCACCCCTGTAAATGCC |
|  |  | Reverse | CGCACATAGCTTTTCCACGTTG |
| *Lifr* | Mouse | Forward | CAACAACATGCGAGTGTGGG |
|  |  | Reverse | CACCAGGTGAAAGAGCTGGA |
| *Mcp-1* | Mouse | Forward | AGGTCCCTGTCATGCTTCTG |
|  |  | Reverse | GCTGCTGGTGATCCTCTTGT |
| *Osm* | Mouse | Forward | GTGGCTGCTCAACTCTTCC |
|  |  | Reverse | AGAGTGATTCTGTGTTCCCCGT |
| *Osmr* | Mouse | Forward | GATGTACCCACTAAGCCGCC |
|  |  | Reverse | GAGGACCGTTGAGGTCAAGC |
| *P-selectin* | Mouse | Forward | ACGAGCTGGACGGACCCG |
|  |  | Reverse | GGCTGGCACTCAAATTTACAG |
| *Tnf-α* | Mouse | Forward | GACGTGGAACTGGCAGAAGA |
|  |  | Reverse | GCCACAAGCAGGAATGAGAA |
| *Vcam1* | Mouse | Forward | TGCCGGCATATACGAGTGTG |
|  |  | Reverse | AAACGATCATCCCGATGGCA |
| *α-Sma* | Rat | Forward | CAGAGGGAAGAGTTCAG |
|  |  | Reverse | CTTGGTCTGGAGGAGACCT |
| *Anp* | Rat | Forward | CGGTACCGAAGATAACAGCCA |
|  |  | Reverse | TCACCACCTCTCAGTGGCAA |
| *Gapdh* | Rat | Forward | CGCTCTCTGCTCCTCCTGTTC |
|  |  | Reverse | ATCCGTTGACTCCGACCTTCAC |
| *Osmr* | Rat | Forward | AGCCCACATGACTTGGAAGG |
|  |  | Reverse | TGAAGAGGTAGTTGGCGCTC |
